# APTAnet: an atom-level peptide-TCR interaction affinity prediction model

**DOI:** 10.52601/bpr.2023.230037

**Published:** 2024-02-29

**Authors:** Peng Xiong, Anyi Liang, Xunhui Cai, Tian Xia

**Affiliations:** 1 School of Artificial Intelligence and Automation, Huazhong University of Science and Technology, Wuhan 430074, China; 2 Institute of Pathology, Tongji Hospital, Tongji Medical College, Huazhong University of Science and Technology, Wuhan 430030, China

**Keywords:** Immunotherapy, TCR, Antigen, Natural language processing, Transfer learning

## Abstract

The prediction of affinity between TCRs and peptides is crucial for the further development of TIL (Tumor-Infiltrating Lymphocytes) immunotherapy. Inspired by the broader research of drug-protein interaction (DPI), we propose an atom-level peptide-TCR interaction (PTI) affinity prediction model APTAnet using natural language processing methods. APTAnet model achieved an average ROC-AUC and PR-AUC of 0.893 and 0.877, respectively, in ten-fold cross-validation on 25,675 pairs of PTI data. Furthermore, experimental results on an independent test set from the McPAS database showed that APTAnet outperformed the current mainstream models. Finally, through the validation on 11 cases of real tumor patient data, we found that the APTAnet model can effectively identify tumor peptides and screen tumor-specific TCRs.

## INTRODUCTION

Tumor Infiltrating Lymphocytes (TIL) immunotherapy (Paijens *et al.* 2021) is one of the methods for treating cancer. It involves isolating and purifying T cells from tumor tissue, expanding them through *in vitro* stimulation and cultivation, and then reintroducing them into the patient’s body. This process amplifies the immune response, leveraging the patient’s own immune capabilities to combat the tumor. However, there are as many as 10^15^ different types of T cells in the human body. Different T cells express distinct T Cell Receptors (TCR) on their surfaces. Only T cells with specific TCRs can selectively recognize tumor antigens and carry out targeted cytotoxic activities. These T cells are referred to as tumor-specific T cells. Therefore, the key challenge in TIL tumor immunotherapy is how to select tumor-specific T cells from a large pool of TIL cells and massively expand their numbers.

In the immune response, tumor antigens are proteolytically cleaved into small peptide molecules (Schumacher and Schreiber 2015). These peptides subsequently bind to the Major Histocompatibility Complex (MHC) and are presented on the cell membrane. The presented peptides are then recognized by the receptor protein TCR on the surface of T cells. Tumor cells recognized by TCR are subsequently eliminated by the immune system. Developing an algorithm to predict the affinity between MHC, peptides, and TCR is crucial for the rapid and precise selection of tumor-specific T cells, further advancing TIL immunotherapy. This will significantly reduce the time and cost required for immune testing experiments and greatly complement experimental methods (Desai and Kulkarni-Kale 2014).

Early methods for predicting TCR-peptide affinity were primarily based on TCR sequence similarity. TCRGP (Jokinen *et al.* 2021) used Gaussian process regression for prediction, while GLIPH (Glanville *et al.* 2017) and TCRdist (Dash *et al.* 2017) measured similarity by defining weighted distances between different TCRs based on different similarity metrics. TCRtopo (Bi *et al.* 2019) constructed a TCR similarity network using DeepWalk (Perozzi *et al.* 2014), and DeepTCR (Sidhom *et al.* 2021) utilized a variational autoencoder to learn implicit representations of TCR sequences and perform TCR clustering. Subsequently, with the introduction of CNN (Jurtz *et al.* 2018) and LSTM (Springer *et al.* 2020) methods, ImRex (Luu *et al.* 2021) transformed sequence features into interaction graphs and applied CNN for prediction. BiAttCNN (Bi *et al.* 2022) combined bidirectional LSTM and attention mechanisms to comprehensively extract key information from amino acid sequences. pMTnet (Lu *et al.* 2021), on the other hand, utilized extensive PMI data and unsupervised TCR data for pre-training and fine-tuning with limited PTI data to enhance generalization. TITAN (Weber *et al.* 2021) proposed a bimodal attention network that extensively learned the binding of protein receptors and ligands and significantly improved performance through transfer learning with PTI data. However, these methods did not fundamentally address the issue of data scarcity and did not fully harness the potential of deep learning and natural language processing (NLP). There is still significant room for improvement in model architecture and training in this context.

Here we introduce a neural network algorithm called APTAnet (Atomic-level Peptide TCR Attention network). This algorithm uses an atomic-level cross-attention mechanism to simulate the interaction process between peptide-TCR sequences and predict interaction affinity. We utilize the ProtBERT model to encode the TCR amino acid sequences and the ChemBERTa2 (Ahmad *et al.* 2022) model to encode the peptide amino acid sequences. Additionally, we employ a cross-attention mechanism to simulate the interaction recognition process between the ligand and receptor sequences. We evaluate the performance of APTAnet on a dataset curated from the McPAS database and a real tumor dataset, demonstrating excellent performance exceeding existing methods.

## MATERIALS AND METHODS

### Data collection

In this study, a total of 45,120 human TCR β sequences were downloaded from the VDJdb database (https://vdjdb.cdr3.net/). These TCR sequences were assigned to 215 peptides. Additionally, all human TCR β sequences related to COVID-19 were downloaded from the ImmuneCODE database (https://www. adaptivebiotech.com/immunecode/), totaling 154,320 TCRs assigned to 289 peptides. The VDJdb database was merged with the ImmunoCODE dataset, retaining only the PTI data related to human MHC I. Furthermore, to limit differences in sequence lengths, we restricted the amino acid length of TCR CDR3β sequences to be between 10 and 20 and the amino acid length of peptide sequences to be between 8 and 14 to retain the majority of valid data.

Next, the data was complemented using the gene reference sequences and VDJ gene information provided by the IMGT database (https://www.imgt.org). Having full-length TCR β sequences provides richer protein context information, which is beneficial for subsequent sequence embedding. Regarding peptides, we used the RDKit Python toolkit for cheminformatics (https://rdkit.org/) to convert peptide sequences into typical SMILES sequences. To ensure the uniformity of pairing relationships, we adopted a sampling approach inspired by Moris *et al.* (Moris *et al.* 2021). Peptides with fewer than 15 TCR pairings were filtered, and downsampling was applied to limit each peptide to a maximum of 400 TCR pairings. Finally, a total of 25,675 pairs of PTI data were obtained, including 23,143 unique TCR sequences and 244 unique peptide sequences.

### Negative sample generation

In the database, output labels are continuous affinity values. To reduce the complexity of the problem and account for potential affinity bias in experimental measurements, we disregarded the continuous affinity values and transformed the regression problem into a binary classification problem. All data stored in the database is labeled as positive data, while negative samples are generated manually.

We selected random mismatch (Fischer *et al.* 2020) as the method for generating negative data. Compared to adding TCR libraries from other sources, the random mismatch approach not only limits the overestimation of model performance but also balances the number of negative and positive samples, thus avoiding dataset class imbalance. To reduce the interference from false negative data, we further refined the data generation method to minimize the similarity between the sampled TCR and the real TCR sequences. The steps for generating negative samples are as follows:

(1) Under specific random numbers, uniformly sample a sequence $ {p}_{i} $ from the peptide set P. It is known that in the positive samples, the set of all TCRs paired with $ {p}_{i} $ is $ \left\{{t}_{j}\right\} $.

(2) Sample a sequence $ {t}_{i} $ from the TCR set *T* in such a way that $ {t}_{i} $ is not an element of $ \left\{{t}_{j}\right\} $.

(3) Calculate the average edit distance *s* between $ {t}_{i} $ and the CDR3 sequences in $ \left\{{t}_{j}\right\} $, using the formula as follows. If the distance *s* is greater than 5, then consider ($ {p}_{i} $, $ {t}_{i} $) as a negative sample.

$ \text{s}=\frac{{\displaystyle\sum }_{j\;=\;1}^{\left|T\right|}D\left({t}_{i},{t}_{j}\right)}{\left|T\right|} , $

where |*T*| represents the number of sequences in the set, and D ($ {t}_{i} $, $ {t}_{j} $) represents the edit distance between sequences $ {t}_{i} $ and $ {t}_{j} $, which is the minimum number of editing operations required to transform one sequence into the other.

(4) Repeat Steps 1 through 3 until the number of negative samples and positive samples are equal.

Through this process, after combining positive and negative samples, the training dataset contained a total of 51,350 pairs of data, with 50% being positive samples and 50% being negative samples.

### Data augmentation

For deep learning models, achieving good generalization on complex problems requires ensuring both the quantity and quality of the dataset. However, due to the limitations of the PTI dataset, the number of high-quality pre-processed data falls far short of what is required for PTI prediction. Research indicates that using data augmentation strategies can mitigate this issue (Wu *et al.* 2021). Data augmentation enriches the diversity of input data, thereby improving the model’s performance.

In this study, SMILES (Weininger *et al.* 1989) sequences data augmentation was implemented using the RDkit library. First, the original SMILES sequences were converted into corresponding molecular objects. Then, based on the molecular structure of the objects, atom identifiers were obtained. Subsequently, the order of atom identifiers was randomly shuffled, and the molecules were renumbered to generate new SMILES sequences. This process was repeated until the desired augmentation level was reached.

SMILES sequence data augmentation can help alleviate the problem of limited antigen diversity in peptides. By greatly expanding the number of peptide sequences through augmentation, it significantly increased the diversity of peptides in the dataset, enriched the input space, and enhanced the model’s generalization ability.

### Data set split

Due to the limited diversity of peptide data in the current dataset, the model is highly prone to overfit when it comes to recognizing entirely new peptide sequences. Therefore, our research was focused on predicting binding between known antigen peptides and any TCR within a specified collection. During data splitting, a “soft split” approach was used, allowing peptides to be distributed randomly, while ensuring that each type of TCR can only appear in either the training set or the test set.

In this study, a 10-fold cross-validation was employed to evaluate the model. A “soft split” ensured that each type of TCR was only restricted to the same fold, while peptides could be randomly distributed in each fold. During each training iteration, nine of the folds were used as the training set, and one fold was used as the test set.

### Pre-training data set

The pre-training data set used in this study was obtained from the BindindDB pre-processed data set provided by Born *et al*. (https://ibm.biz/active_site_data). To ensure the similarity in length distribution between the DPI data and PTI data and avoid significant differences in sequence lengths, ligands with SMILES sequences longer than 300 and proteins with amino acid sequences longer than 512 were excluded from the DPI data. The subsequent processing was similar to the PTI data set, where all data in the dataset were treated as positive samples, and negative samples were generated through random mismatches. In the end, the DPI data set contained 177,225 ligand molecules and 1679 protein receptors, totaling 409,859 pairs of data, with 90% used for pre-training and 10% used to validate model performance.

### Independent data set

We tested the model using an independent test set generated from the McPAS database (http:// friedmanlab.weizmann.ac.il/McPAS-TCR/). To create this test set, we initially excluded TCR sequences that were already present in the VDJdb, ensuring the test set’s heterogeneity. Subsequently, we categorized the independent test set into two groups based on peptide presence in the training set: SP (Seen Peptide) and UP (Unseen Peptide). In the SP category, peptide sequences had been part of the training sets for all models, while the UP category included peptide sequences not present in any model’s training set. Finally, after applying data filtering and generating negative samples, the SP test set contained 5232 data pairs, and the UP test set contained 928 data pairs.

### K-NN baseline

To better assess the general performance of the PTI problem, we employed a K Nearest Neighbor (KNN) model based on sequence similarity as the baseline classifier. We used the sum of normalized Levenshtein distances between amino acid sequences of peptides and TCRs as the distance metric between samples.

For the dataset $ \mathcal{D}={\left\{{p}_{i},{t}_{i},{a}_{i}\right\}}_{i\;=\;1}^{N} $, where $ {p}_{i} $ and $ {t}_{i} $ are amino acid sequences of peptides and TCRs, for any two sample points $ \left\{{p}_{i},{t}_{i}\right\} $ and $ \left\{{p}_{j},{t}_{j}\right\} $, their distance metric is defined as follows:

$ D\left({e}_{i},{t}_{i},{e}_{j},{t}_{j}\right)=\frac{Lev\left({e}_{i},{e}_{j}\right)}{\left|{e}_{j}\right|}+\frac{Lev\left({t}_{i},{t}_{j}\right)}{\left|{t}_{j}\right|} , $

where $ \left|\cdot \right| $ is sequence length and $ Lev\left(\cdot ,\cdot \right) $ is the Levenshtein distance.

Model performance was evaluated for all odd values of k (1 < *k* < 20). When *k* is greater than or equal to 11, the ROC-AUC and PR-AUC values of the model tend to be consistent. We chose KNN with *k* = 19 as the baseline model to assess the benchmark classification results for the PTI problem.

### APTAnet model

**Figure 1:**
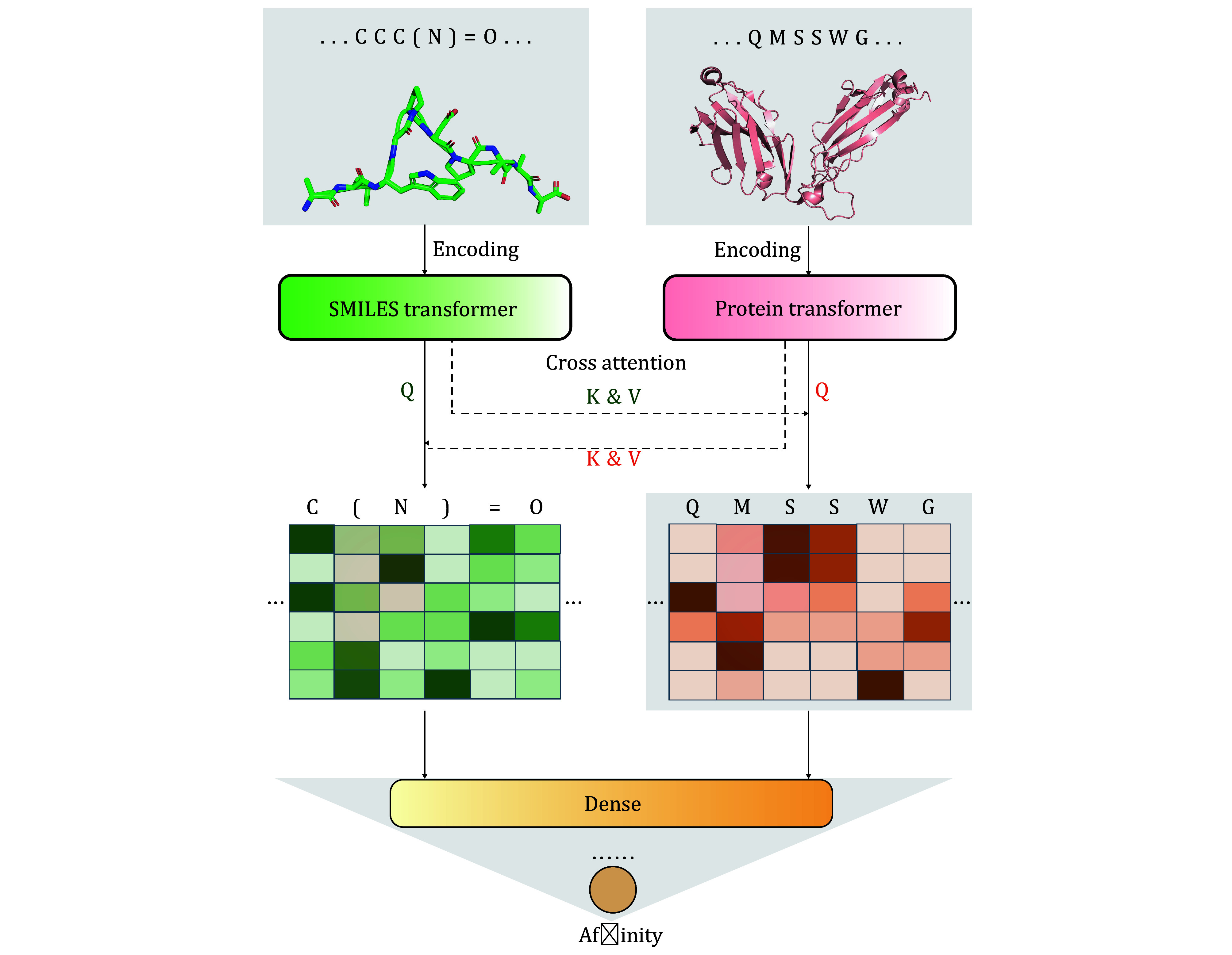
APTAnet architecture. APTAnet consists of three main components: the encoding module, the cross-attention module, and the fully connected module. It begins by embedding the peptide and TCR sequences separately using the encoding module. Then, it allows interaction between the encoded vectors using cross-attention, and finally, the fully connected module extracts relationships between the features to predict the affinity score

Figure 1 displays the overall structure of APTAnet, the model simulates the interaction process between peptide-TCR sequences and predicts the affinity of their interaction. APTAnet consists of three main components: the encoding module, the cross-attention module, and the fully connected module.

#### The encoding module

In the encoding module, we use pre-trained Transformer-based models to embed the SMILES sequences and amino acid sequences. The encoding module parameters are as shown in Table 1. For encoding amino acid sequences, we utilize the ProtBERT pre-trained model (https://huggingface.co/Rostlab/prot_BERT_bfd). ProtBERT is based on BERT, with 30 layers of Transformer structures, each having 16 attention heads. Each amino acid is embedded into a 1,024-dimensional vector. For encoding SMILES sequences, we use the ChemBERTa2 pre-trained model (https://huggingface.co/DeepChem/ChemBERTa-77M-MTR). ChemBERTa2 is based on RoBERTa, which is an optimized model based on BERT. It deploys 12 layers of Transformer structures, each with six attention heads. Each SMILES symbol is embedded into a 384-dimensional vector. In the encoding process, a tokenizer first segments the input sequences at the character level. The pre-trained model adds a special token [CLS] (classification) at the beginning of each sequence, and the vector obtained from embedding this symbol can represent the semantic information of the entire sequence. In addition, multiple [PAD] (padding) tokens are added to the end of each sequence to pad it to a fixed length, *L*, ensuring consistency for sequences of different lengths. Finally, the tokenized vectors and positional encodings of the sequence are input into the model. The model embeds each character into a D-dimensional vector and concatenates them to form the encoding of the entire sequence.

**Table 1:** Encoding module parameters

Sequence type	Amino acid (AA)	SMILES
Model	ProtBERT	ChemBERTa2
Attention Heads	16	12
Transformer Layers	30	6
Model Parameters	419,931,136	34,274,400
Padding Length(L)	512	320
Embedding Dimension D	1024	384
Training Data	4 × 10^11^	8 × 10^7^

#### Cross-attention module

**Figure 2:**
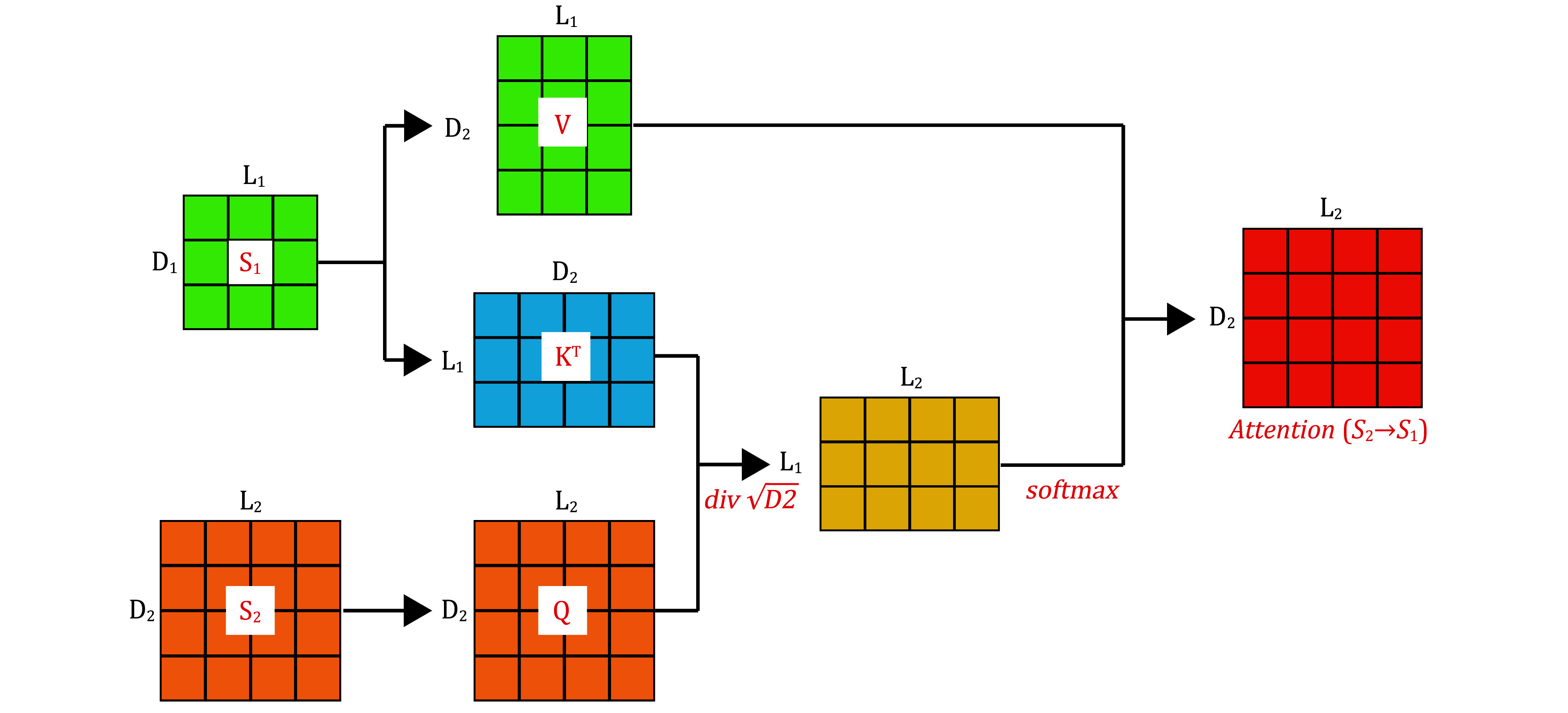
Cross-Attention involves two input matrices, $ {S}_{1} $ and $ {S}_{2} $, which have different dimensions. It is necessary to project Q, K, and V to different attention spaces using distinct projection matrices $ {W}_{Q} $, $ {W}_{K} $, and $ {W}_{V} $. Afterward, attention weights are calculated through dot-product operations and softmax activation functions. The final output matrix has the same dimensions as the receptor matrix $ {S}_{2} $

Taking inspiration from the self-attention (Vaswani *et al.* 2017) mechanism, we apply paired cross-attention mechanisms to the PTI problem. By using two paired cross-attention layers, attention is computed for the receptor (T) based on the ligand (P) to calculate the attention (P→T), and vice versa, where the ligand (P) becomes the source to calculate attention on the receptor (T) to obtain attention (T→P). This enables the receptor to utilize information from the ligand to learn the importance of each symbol in the input sequence (Fig. 2). The formula for calculating cross-attention weights is as follows:

$ Attention\;({S}_{2}\to {S}_{1})=softmax\left(\frac{\left({S}_{2}{W}_{Q}\right){\left({S}_{1}{W}_{K}\right)}^{{\mathrm{T}}}}{\sqrt{{D}_{2}}}\right){S}_{1}{W}_{V} , $

where $ {S}_{1}\in {\mathbb{R}}^{{L}_{1}\;\times\; {D}_{1}} $ represents the input ligand sequence, $ {L}_{1} $ is the length of the ligand sequence, and $ {D}_{1} $ is the feature dimension of the ligand encoding. $ {S}_{2}\in {\mathbb{R}}^{{L}_{2}\;\times \;{D}_{2}} $ represents the input receptor sequence, where $ {L}_{2} $ is the length of the receptor sequence, and $ {D}_{2} $ is the feature dimension of the receptor encoding. $ {W}_{Q}\in {\mathbb{R}}^{{D}_{1}\;\times\; {D}_{1}} $, $ {W}_{K}\in {\mathbb{R}}^{{D}_{1}\;\times \;{D}_{2}} $, and $ {W}_{V}\in {\mathbb{R}}^{{D}_{1}\;\times\; {D}_{2}} $ are the projection matrices.

Considering the multi-head attention mechanism, we use eight parallel cross-attention heads to construct an attention layer, and the calculation formula is as follows:

\begin{equation*}\begin{split} &
MultiHead-Attention\left({S}_{2}\to {S}_{1}\right)\\&
= Concat\left(hea{d}_{1},hea{d}_{2},\dots ,hea{d}_{h}\right){W}^{\,O}. 
\end{split}\end{equation*}

#### Fully connected module

The fully connected module initially concatenates the interaction vectors obtained from the cross-attention module and normalizes the data distribution through batch normalization. Subsequently, it applies multiple Dense modules for nonlinear transformations of the data to extract relationships between features. Finally, it maps the input to a value between 0 and 1 using the Sigmoid function, resulting in the output affinity score.

Each Dense module consists of four layers. The first layer is the Linear layer, which performs a linear transformation from a high-dimensional vector to a lower-dimensional one. The second layer is the Batch Normalization layer, which standardizes the input data distribution of each layer by normalizing each small batch of samples, making it closer to a standard normal distribution. The introduction of Batch Normalization can effectively alleviate the vanishing gradient problem, accelerate model training, and suppress overfitting.

The third layer is the Activation layer, which introduces non-linearity through an activation function, enhancing the neural network’s expressive power. We employed the ReLU activation function here. The fourth layer is the Dropout layer, which, during forward propagation, deactivates each neuron with a certain probability (p), preventing the model from relying too much on specific local features and, to some extent, achieving regularization.

### Model training

Since PTI is a binary classification problem, the loss function used is binary cross-entropy (BCE), and the calculation formula is as follows:

$ BCELoss=-\left(y\mathrm{log}\left(p\right)+\left(1-y\right)\mathrm{log}\left(1-p\right)\right) , $

where *y* is the true label and *p* is the predicted affinity score. Cross-entropy is a good measure of the difference between the true probability distribution and the predicted probability distribution.

We use the AdamW optimizer (Loshchilov and Hutter 2017) to update the model parameters during training, which adjusts model parameters based on the gradients of the loss function. AdamW is known for its excellent performance in deep learning, particularly in tasks such as natural language processing and computer vision, and can yield better results.

In terms of the learning rate adjustment strategy, we employ a OneCycleLR (Smith and Topin 2017) learning rate adjustment strategy based on the cosine function. Throughout the training process, the learning rate first increases and then decreases, forming an approximate cosine curve.

The training process of the APTAnet model is divided into two stages: pre-training and fine-tuning. In the pre-training stage, affinity data between drugs and proteins from the DPI dataset are used to learn a broad range of ligand-receptor interactions. By learning from a large amount of ligand data, the input space of the SMILES channel is significantly enriched, enhancing the model’s generalization capability to different data. In the fine-tuning stage, the model is fine-tuned using PTI data. After converting peptides into SMILES sequences, the PTI task can be considered a specific subtask of DPI, allowing for transfer learning. However, due to the differences in SMILES data between DPI and PTI, when importing the pre-trained model for fine-tuning, a “Semi-Frozen” approach is employed. This means that the model weights for the Protein channel are frozen, and only the model for the SMILES channel and the fully connected modules are trained. This approach allows for better adaptation of the model to the specific requirements of the PTI task while leveraging the knowledge gained during pre-training.

## RESULTS

### Overview of approach

**Figure 3:**
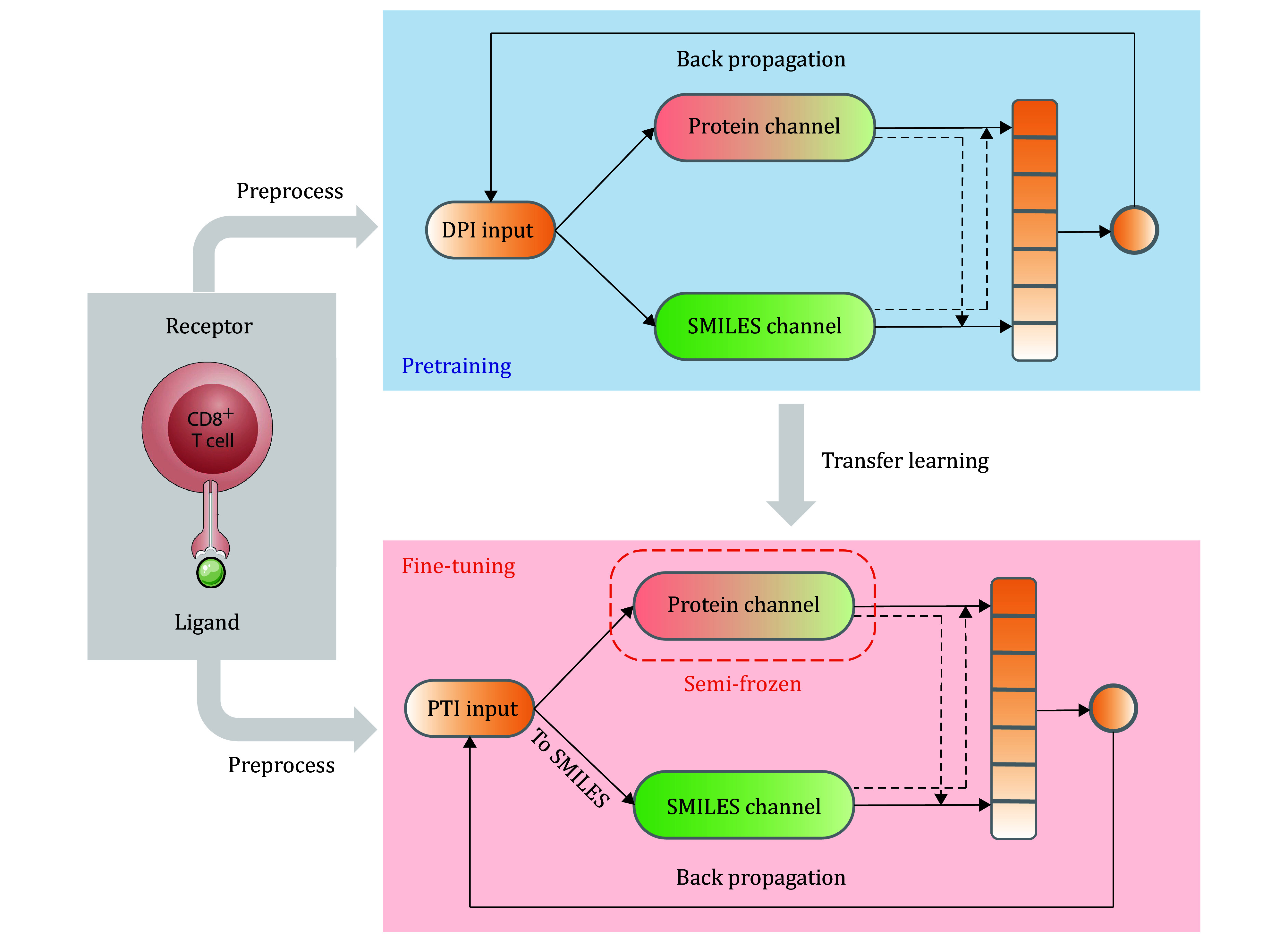
APTAnet architecture. The training process of the APTAnet model is divided into two stages: pre-training and fine-tuning. In the pre-training stage, affinity data between drugs and proteins from the DPI dataset are used to learn a broad range of ligand-receptor interactions. In the fine-tuning stage, the model is fine-tuned using PTI data

APTAnet model was trained on data from the DPI dataset, which was obtained from BindingDB, containing 177,255 ligand molecules and 1679 protein receptors (see the MATERIALS AND METHODS section). After learning the general binding modes of protein receptors and drug ligands, the model is fine-tuned using PTI data. We downloaded TCR data from VDJdb, performed data transformation, and applied filtering, obtaining 23,143 unique TCRs and 244 unique peptides (see the MATERIALS AND METHODS section). We adopted a “Semi-Frozen” approach, which involves freezing the model weights of the protein channel and training only the model weights of the SMILES channel and the fully connected module (Fig. 3).

### Performance analysis of different encoding methods

**Figure 4:**
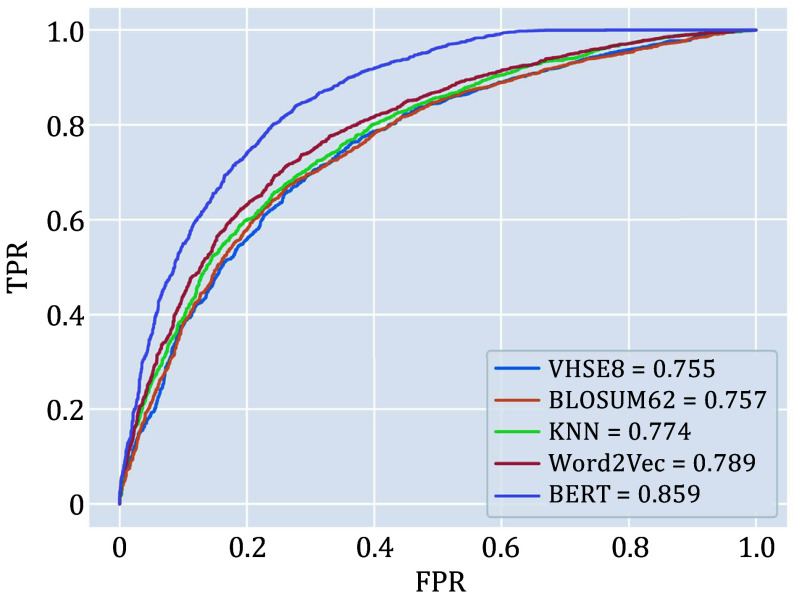
ROC curves for different encoding methods

To determine which sequence encoding method works best, we first compared the performance of different encoding methods (Table 2). The BERT pre-trained model achieved the highest ROC-AUC (0.859), ahead of VHSE8 (ROC-AUC = 0.755), BLOSUM62 (ROC-AUC = 0.757), Word2Vec (ROC-AUC = 0.789), and K-NN baseline (ROC-AUC = 0.774) (Fig. 4). ChemBERTa2 and ProtBERT have been trained on vast amounts of sequence language, allowing them to learn the representation of amino acids and SMILES language expressions comprehensively. They can capture the semantics of sequences based on context and extract key sequence features. Therefore, in downstream classification predictions, encoding methods based on BERT pre-trained models tend to provide superior performance compared to other encoding methods.

**Table 2:** Encoding method table

TCR encoding method	Peptide encoding method	ROC-AUC
Amino acid(VHSE8)	Amino acid(VHSE8)	0.755
Amino acid(BLOSUM62)	Amino acid(BLOSUM62)	0.757
Amino acid(ProtVec)	SMILES(SMILESVec)	0.789
Amino acid(ProtBERT)	SMILES(ChemBERTa2)	0.893

**Figure 5:**
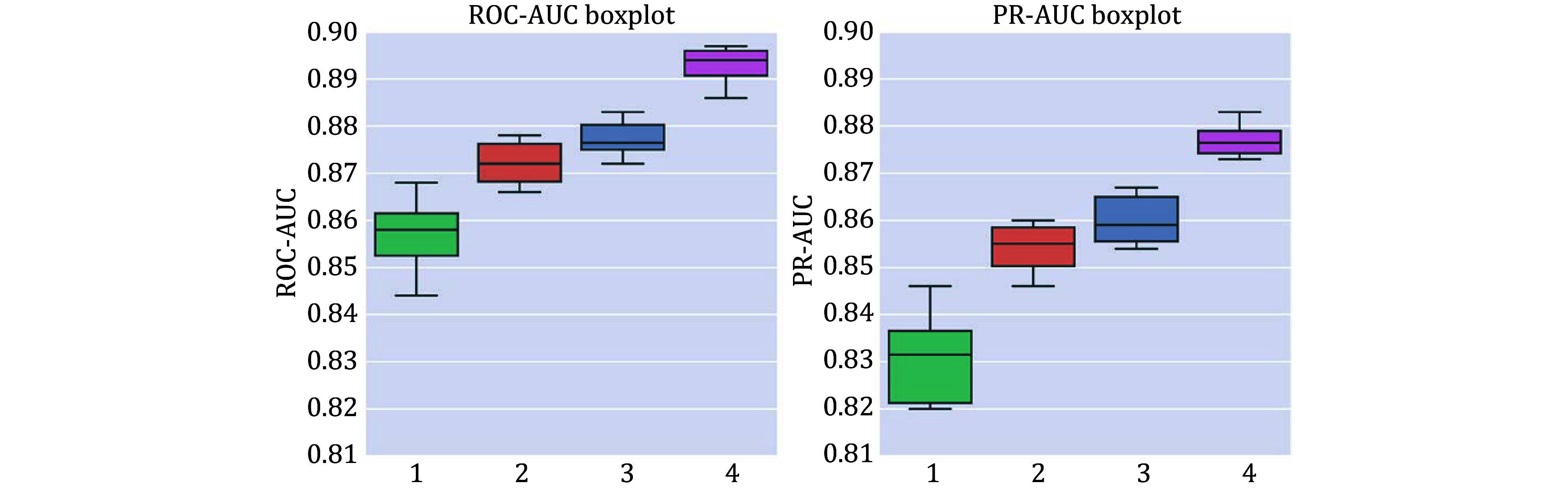
Box plots of ten-fold cross-validation ROC-AUC (left) and PR-AUC (right) for different training modes. Group 1 is APTAnet without pre-training, Group 2 is APTAnet + Pretrain, Group 3 is APTAnet + Pretrain + Semifrozen, and Group 4 is APTAnet + Pretrain + Semifrozen + Augmentation

We next compared the performance of different training strategies (Table 3). We incrementally added three training strategies on top of training directly on the PTI dataset: DPI pre-training, weight freezing, and data augmentation. Without pre-training, “APTAnet” achieved average ROC-AUC and PR-AUC values of 0.856 and 0.831. After adding the “Pretrain” approach, the model’s average ROC-AUC and PR-AUC improved to 0.872 and 0.854. Following that, with the protein channel weights frozen in the transfer learning stage, the model’s average ROC-AUC and PR-AUC increased to 0.877 and 0.860. Finally, with the introduction of “Augmentation”, the model’s performance saw a substantial boost, with average ROC-AUC reaching 0.893 and mean PR-AUC reaching 0.877 (Fig. 5). These results indicate that representing ligand peptides using SMILES notation is superior and that utilizing pre-training and data augmentation can lead to significant improvements in the model’s performance.

**Table 3:** Training strategy table

Training mode	ROC-AUC	PR-AUC
APTAnet	0.856 ± 0.013	0.831 ± 0.015
APTAnet + Pretrain	0.872 ± 0.008	0.854 ± 0.009
APTAnet + Pretrain + Semifrozen	0.877 ± 0.007	0.860 ± 0.009
APTAnet + Pretrain + Semifrozen + Augmentation	0.893 ± 0.008	0.877 ± 0.007

Finally, we replaced the cross-attention module with self-attention module and concatenation, and compared their performance (Table 4). APTAnet with cross-attention performed best (ROC-AUC = 0.893), ahead of self-attention (0.857) and concatenation (0.791). Cross-attention can effectively capture the complex relationship between TCR and peptide. This attention mechanism allows the model to focus on the parts of the sequences with significant mutual influence when processing TCR and peptide sequences, thereby better capturing the associations between them. Additionally, cross-attention also aids the model in better understanding and leveraging the heterogeneity of information between the TCR and peptide.

### Outstanding performance of APTAnet compared to existing tools

**Figure 6:**
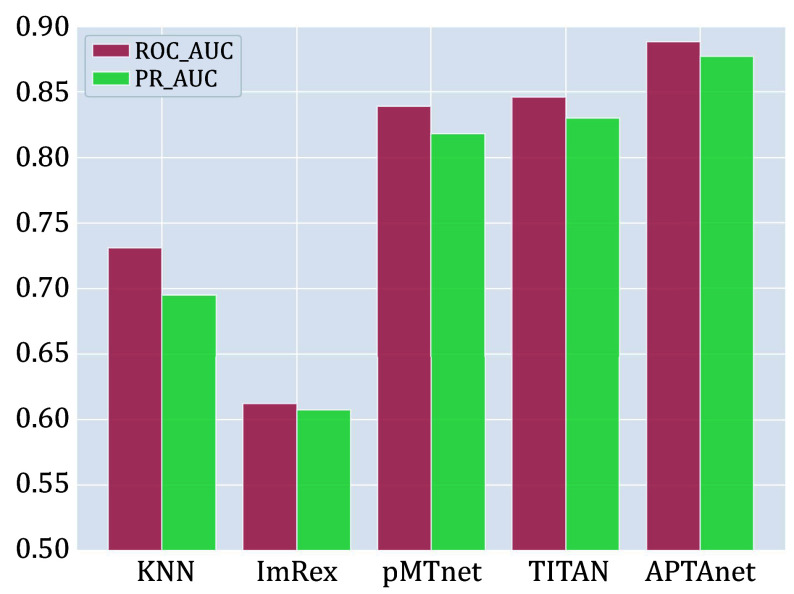
Performance comparison of multiple tools in the SP test set. The bars display the values of ROC-AUC (red) and PR-AUC (green) in different models

**Figure 7:**
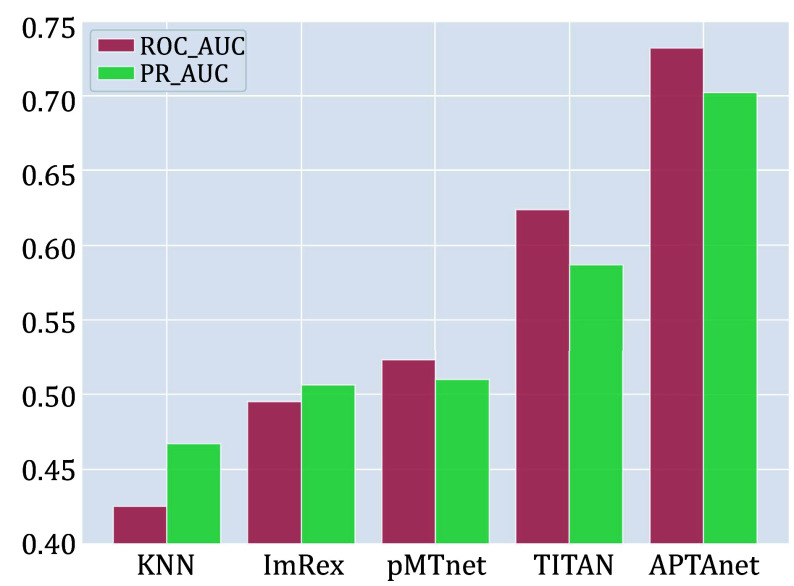
Performance comparison of multiple tools in the UP test set. The bars display the values of ROC-AUC (red) and PR-AUC (green) in different models

We next compared APTAnet with current leading models on independent and publicly available datasets. The test set was generated from the McPAS database and divided into two groups: SP (Seen Peptide) and UP (Unseen Peptide) (see the MATERIALS AND METHODS section). The SP data set contained 5232 data pairs, and the UP data set contained 928 data pairs. On the SP data set, APTAnet achieved the highest ROC-AUC (0.888) and PR-AUC (0.876), ahead of TITAN (ROC-MUC = 0.846 and PR-AUC = 0.829), pMTnet (ROC-MUC = 0.835 and PR-AUC = 0.817), ImRex (ROC-AUC = 0.612 and PR-AUC = 0.602), and K-NN baseline (ROC-AUC = 0.731, PR-AUC = 0.705) (Fig. 6). On the UP data set, APTAnet still achieved the highest ROC-AUC (0.732) and PR-AUC (0.701), ahead of TITAN (ROC-MUC = 0.624 and PR-AUC = 0.587), pMTnet (ROC-MUC = 0.521 and PR-AUC = 0.512), ImRex (ROC-AUC = 0.494 and PR-AUC = 0.506), and K-NN baseline (ROC-AUC = 0.424 and PR-AUC = 0.468) (Fig. 7). In summary, APTAnet outperformed all other models in our analysis, indicating its competitive advantage in predicting known peptide data and strong generalization ability to entirely new peptides.

### Effective tumor peptide identification with APTAnet in real tumor data

**Figure 8:**
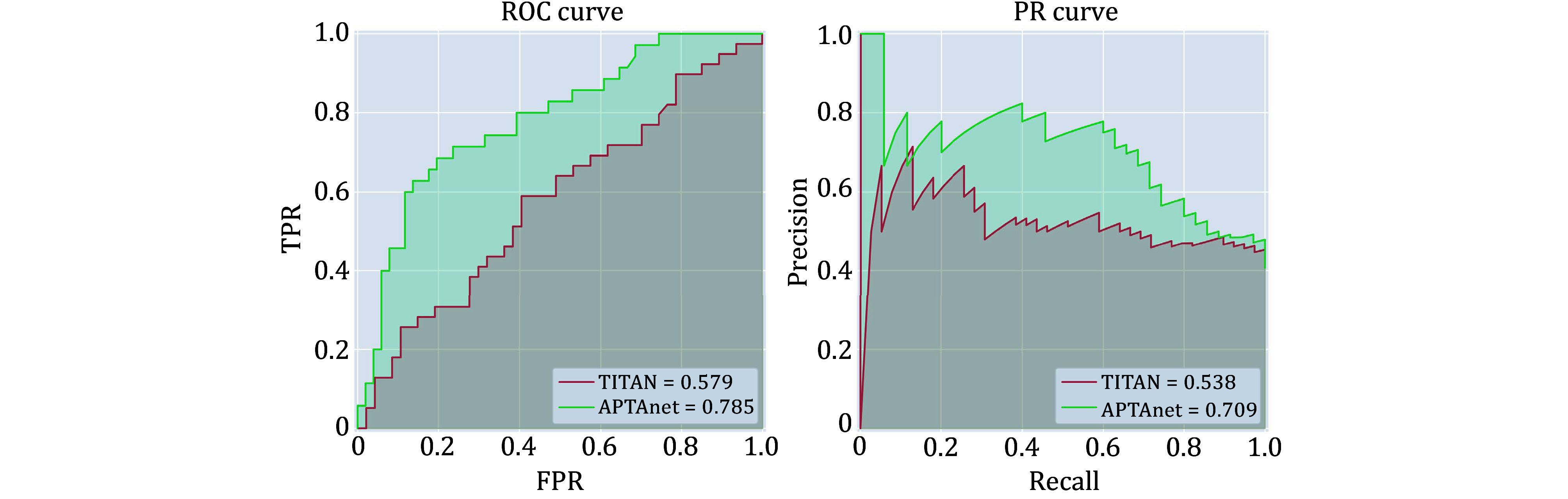
Performance comparison between APTAnet and TITAN in real cancer data. The green line represents APTAnet, and the red line represents TITAN

We further assessed the APTAnet’s performance on the real tumor data. The tumor data set was collected from 11 tumor patients with different cancer types from cancer immunotherapy clinical research (Hanada *et al.* 2022; Lowery *et al.* 2022; Peri *et al.* 2021; Tran *et al.* 2015), comprising a total of 86 data pairs. The APTAnet model (ROC-AUC = 0.785, PR-AUC = 0.709) exhibits significantly better predictive performance compared to the TITAN model (ROC-AUC = 0.579, PR-AUC = 0.538) (Fig. 8). These results indicate that APTAnet possesses excellent generalization capabilities for unknown peptides and the identification of important PTI binding sites.

### TCR sequence generalization analysis

**Figure 9:**
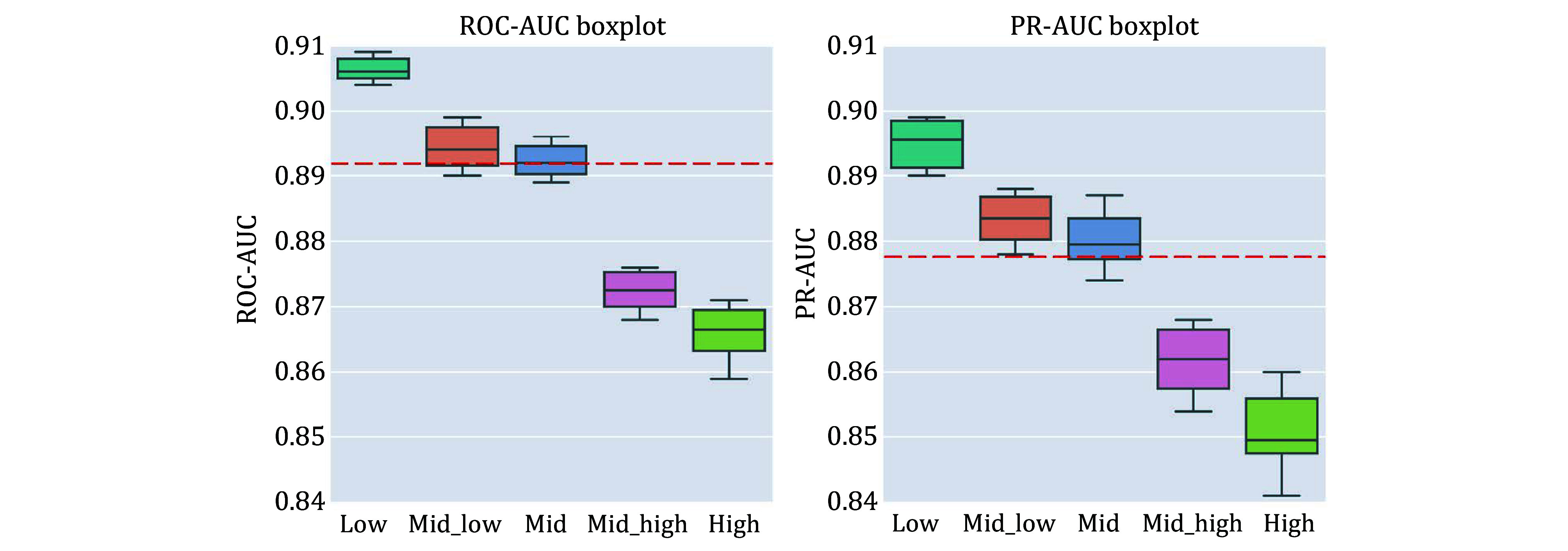
Box plots of ten-fold cross-validation ROC-AUC (left) and PR-AUC (right) for different similarity distances. The red dashed line represents the mean of the overall results

We next examined the model’s generalization ability. We calculated the edit distance between each TCR in the test set and all TCRs in the training set (see the MATERIALS AND METHODS section) and classified the test set into five groups: “low”, “mid-low”, “mid”, “mid-high”, and “high” based on the magnitude of the inter-group distance. APTAnet indeed performs better in predicting TCRs with higher similarity (Fig. 9). The “low”, “mid-low”, and “mid” groups perform above the overall mean (ROC-AUC = 0.893, PR-AUC = 0.877), with the highest similarity “low” group achieving ROC-AUC and PR-AUC values of 0.907 ± 0.003 and 0.895 ± 0.005, respectively. The “mid-high” and “high” groups perform below the overall mean, but even the lowest similarity “high” group still exhibits relatively high performance (ROC-AUC = 0.866 ± 0.008, PR-AUC = 0.851 ± 0.010). This indicates that APTAnet has essentially learned the pairing features of PTIs and has a high degree of generalization for different TCRs, allowing it to recognize sequence patterns of different TCRs effectively.

### Attention mechanism interpretability

Finally, we sought to explain the principles of APTAnet using attention mechanisms. Through statistical analysis of attention results across numerous sequences, it was observed that the average variance of attention scores at the same position for different TCRs is 9.3 × 10^−5^, while for different peptides, it is 1.2 × 10^−4^ on average. Particularly, the TCR CDR3 region exhibits a significantly higher average variance of 3.5 × 10^−4^. This indicates that APTAnet demonstrates certain attention preferences for specific positions in the sequences, and the model can adaptively adjust its focus points for different sequences.

**Figure 10:**
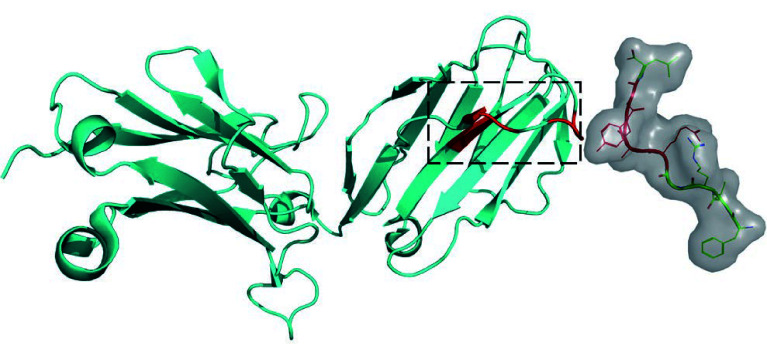
Structure of LC13 TCR in complex with HLAB8-EBV peptide complex (1MI5). The left side in cyan represents TCR β, while the right side in green represents the peptide. The red-highlighted regions indicate the key interaction sites confirmed based on the three-dimensional structure of the PTI. The black-boxed area on the TCR marks the position of CDR3 β, a region known for its high variability and widely recognized for its interaction with peptides

To gain a more intuitive understanding of the biological significance of APTAnet’s attention mechanism, we employed experimentally validated protein complexes (PDB ID: 1MI5) to visualize the atomic-level attention for peptides and the amino acid-level attention for TCRs (Fig. 10). In this context, the amino acid sequence of the peptide is “FLRGRAYGL”, and the CDR3 β sequence of the TCR is “CASSLGQAYEQYF”. According to the predictions from the APTAnet model, the affinity score between them is 0.857, indicating a strong interaction.

**Figure 11:**
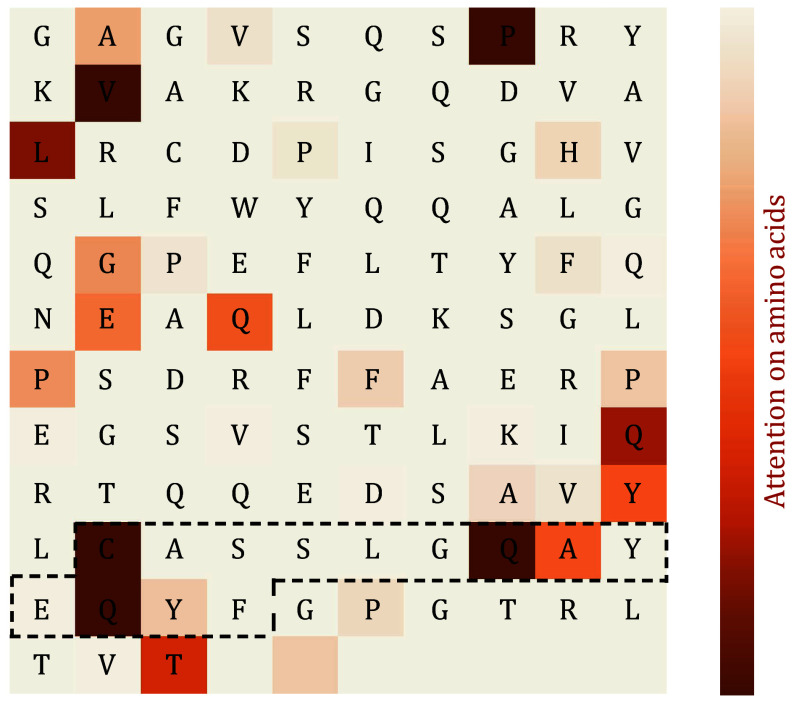
Amino acid-level attention mechanism analysis. The amino acid sequence is horizontally arranged in the matrix, and the symbols in the middle of the matrix represent the corresponding amino acid abbreviations (blank areas indicate the subsequent PAD region of the sequence). The black-boxed region on the TCR corresponds to the CDR3 area. The colors filled in the heatmap represent the strength of attention, with darker colors indicating stronger attention to specific amino acids

We next analyzed the attention scores on amino acids of the TCR sequence. The high-attention points identified by the model in the CDR3 region exhibit a high degree of consistency with the contact points confirmed through three-dimensional structural analysis. Moreover, 10 out of 133 amino acids have high attention scores, and 4 out of the 10 (C91, Q98, A99, Q101) are located in the PTI binding region of the CDR3. This indicates that the model effectively recognizes the PTI recognition region (*P*-value = 0.0018) (Fig. 11). Although other amino acids not in proximity to the binding sites may also have high attention scores, the model’s predictions are still significant, suggesting these amino acids might play a potential role in PTI interactions.

**Figure 12:**
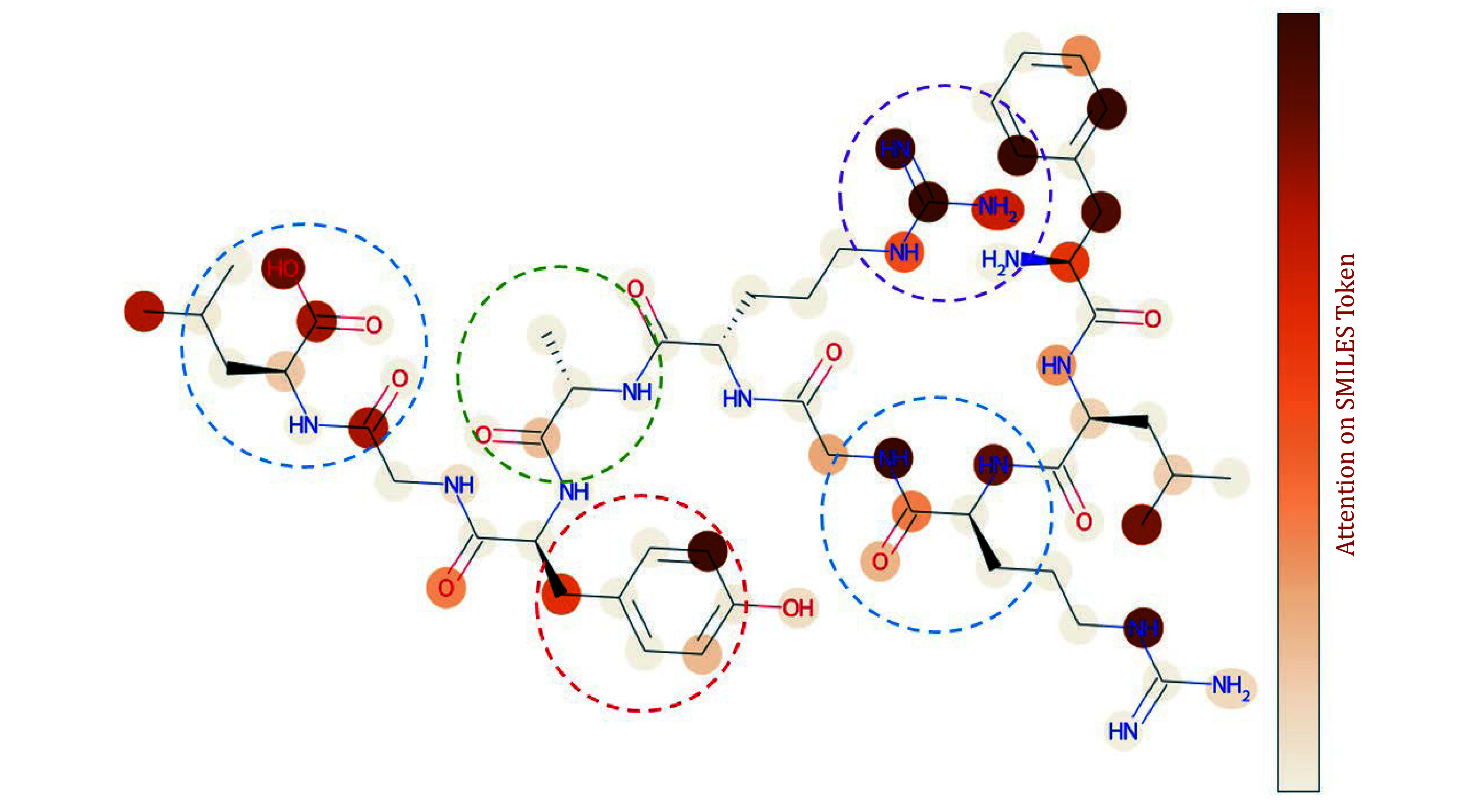
Atom-level attention mechanism analysis. The peptide sequence “FLRGRAYGL” is represented as a corresponding molecular structure diagram, where each node in the molecular graph represents a corresponding atom, the connecting lines represent the chemical bonds between atoms, and the shading of each atom indicates the level of attention that the model places on it in relation to the TCR. The blue boxes represent oxygen-containing functional groups, the red box represents polar amino acids, the purple box represents charged amino acids, and the green box represents nonpolar amino acids

We further analyzed the attention scores at the atomic level of the peptide. There is a high degree of consistency between the key atoms confirmed through structural analysis and the high-attention atoms predicted by the model. We found that, on the peptide’s backbone, the model exhibits higher attention to oxygen-containing groups, such as carboxyl groups and peptide bonds. On the peptide’s side chains, the model shows higher attention to polar amino acids, like tryptophan, and charged amino acids, such as arginine, while exhibiting lower attention to non-polar amino acids, such as glycine (Fig. 12). This indicates that the model is capable of recognizing and focusing on key chemical features related to the peptide-TCR interaction.

Overall, these results indicate that APTAnet has the capability to recognize essential atomic groups or amino acids involved in PTI processes.

## DISCUSSION

The immune response to tumors forms the foundation of TIL immunotherapy. Currently, traditional immunological experimental techniques cannot meet the demands of large-scale applications of TIL immunotherapy. Therefore, the development of a PTI affinity prediction algorithm to enable the rapid and accurate screening of tumor-specific T cells is crucial for the further advancement of TIL immunotherapy. However, the scarcity of data and the vast sequence matching space make this task challenging. Previous models have been limited by data and methods, achieving only limited performance.

This study draws inspiration from the DPI problem and NLP methods, proposing APTAnet — a model for atomic-level PTI affinity prediction. APTAnet provides an excellent solution for accurate PTI affinity prediction and the application of TIL immunotherapy. Due to variations in experimental techniques, conditions, and sample sources, different experimental results may exhibit potential numerical biases in affinity values. Therefore, to reduce batch effects in experiments and simplify the complexity of the problem, we binarize the continuous regression values. The results obtained in this manner are more interpretable and practical. A drawback of binary tasks is the inability to compare the binding strengths between different TCR-peptide pairs.

The superior performance of APTAnet may be attributed to the design of a model training method that enriches the training data to mitigate the limitations of existing PTI data. This method treats the PTI problem as a subtask of the DPI problem, further representing the peptide’s amino acid sequence as an atomic-level, fine-grained SMILES sequence. This approach not only enables data augmentation, enriching the input data space but also leverages the large-scale dataset from BindingDB (Gilson *et al.* 2016) for pre-training. This pre-training extensively learns the binding patterns of proteins and ligands, followed by fine-tuning with PTI data, significantly improving the model’s generalization capability. The predicted results can be validated using MHC multimer technology: This method utilizes multimers that bind to MHC molecules, allowing the binding of multiple T cells specific to the same antigen. This aids in the detection and analysis of T cell responses to specific antigens.

Compared to existing tools, APTAnet has achieved precise predictive performance with limited data. This not only provides valuable support for the application of TIL immunotherapy research but also strongly demonstrates the feasibility of applying NLP and deep learning methods to biological sequences.

The future of PTI research depends on constructing high-quality, large-scale, and standardized PTI affinity databases, proposing more general PTI model methods, and implementing end-to-end tumor affinity prediction methods. The immune response process of tumors mainly involves the interaction of MHC, peptide, and TCR complexes. Designing models that predict all three inputs end-to-end, though more complex, has the potential to further enhance model performance by providing more input information.

In summary, we have designed APTAnet, which can predict the affinity of peptide-TCR binding based on the amino acid sequences of peptides and TCRs at the sequence level. This allows for the screening of potential tumor-specific T cells to assist in TIL cancer immunotherapy.

**Table 4:** Ablation study

Attention Module	ROC-AUC
Cross-attention	0.893
Self-attention	0.857
Concatenation	0.791

## Conflict of interest

Peng Xiong, Anyi Liang, Xunhui Cai and Tian Xia declare that they have no conflict of interest.
